# Evaluation of Alexithymia, Somatosensory Sensitivity, and Health Anxiety Levels in Patients with Noncardiac Chest Pain

**DOI:** 10.1155/2014/896183

**Published:** 2014-05-25

**Authors:** Selma Bozkurt Zincir, Murat Sunbul, Esra Aydin Sunbul, Bahar Dalkilic, Fatma Cengiz, Tarik Kivrak, Erdal Durmus

**Affiliations:** ^1^Erenkoy Training and Research Hospital for Psychiatric and Neurological Disorders, Psychiatry Clinic, Istanbul, Turkey; ^2^Department of Cardiology, Marmara University Faculty of Medicine, Fevzi Çakmak Mahallesi, Mimar Sinan Caddesi, No. 41, Üstkaynarca, Pendik, 34899 Istanbul, Turkey

## Abstract

*Objective.* Noncardiac chest pain (NCCP) is seen more frequently in young population and in these patients loss of function is evolving in social and professional areas. The aim of the study is to evaluate the levels of anxiety and somatic perception in patients with chest pain presenting to cardiology clinic. *Methods.* Fifty-one patients with noncardiac chest pain and 51 healthy controls were included in the study. All participants performed self-report based health anxiety inventory (HAI), somatosensory amplification scale (SAS), and Toronto alexithymia scale (TAS). *Results.* The patient group had significantly higher scores on the SAS, HAI-1, and HAI-T scales compared to controls (*P* < 0.001, *P* = 0.006, and *P* = 0.038, resp.). SAS, HAI-1, and HAI-T scores were significantly higher in female patients than male (*P* = 0.002, 0.036, and 0.039, resp.). There were significant differences in all TAS subscale scores between two groups. Patients, who had total TAS score more than 50, also presented higher levels of health anxiety (*P* = 0.045). * Conclusions.* Anxiety, somatic symptoms, and the exaggerated sense of bodily sensations are common in patients with NCCP. These patients unnecessarily occupy the cardiology outpatient clinics. These negative results can be eliminated when consultation-liaison psychiatry evaluates these patients in collaboration with cardiology departments.

## 1. Introduction

Chest pain is one of the most common medical complaints in general population. Since it may be a warning sign of coronary artery disease (CAD) or myocardial infarction (MI), it is also one of the most frightening pains [1, 2]. Between 52% and 77% of patients presenting to the emergency department and referred for coronary angiography suffer chest pain that is not cardiac in origin and many chest pain patients do not receive a medical explanation for their pain [3–5].

Noncardiac chest pain (NCCP) is defined as a recurrent chest pain that is indistinguishable from ischemic heart pain after a reasonable workup has excluded a cardiac cause. NCCP may report squeezing or burning substernal chest pain, which may radiate to the neck, arms, jaws, and back, and is indistinguishable from cardiac angina [6]. NCCP is sometimes regarded as the sensitive heart because of the higher rate of occurrence and greater pain intensity in this pain population [7, 8]. Early conceptualizations of cardiophobia characterized this syndrome by fears of heart attack and death, suggesting that NCCP patients may focus attention on their heart when experiencing stress and arousal [9–11]. These patients also use more commonly sensory and affective words than patients with ischemic heart disease [9]. NCCP patients view their condition as significantly less controllable and less understandable than those patients whose pains are of cardiac origin [11–13].

Despite a favorable long-term cardiovascular prognosis [12–15], NCCP is a major public health concern that not all NCCP patients have good outcomes [16]. NCCP is associated with impaired daily activities (e.g., work, walking, exercising, and housework), reduced quality of life, and increased occupational and social disability comparable to patients with CAD [17, 18]. Many patients experience worry, anxious preoccupation with heart functioning, and recurrent chest pain which results in increased health care costs due to frequent hospitalizations, emergency department visits, and cardiac catheterizations [7–9, 19].

Due to the diagnostic classification of NCCP as a “diagnosis” of exclusion (i.e., medical rule-outs) rather than diagnostic inclusion, understanding of this syndrome has been slowed and complicated [17]. Modern theoretical models of NCCP are multicausal and emphasize the multidisciplinary nature of the problem [1, 2]. Biological vulnerability, stress, and psychological vulnerability are considered to be leading to anxious apprehension and learned alarm [1, 10, 13–15]. Theories of NCCP are supported by researches showing that psychological factors like anxiety sensitivity, conscious hypervigilance to physical sensations, and alexithymia may be relevant to this medically unexplained syndrome [10, 15, 17, 18].

Moreover, physicians may be reluctant to ask about the psychological symptoms in these patients, as it has been supposed that patients are defensive about symptoms due to mental illness. The present study was designed to determine the health anxiety level and somatic symptom sensitivity in patients referred to a cardiology clinic for their unexplained chest pain. Our second aim was to examine influences of alexithymia and anxiety sensitivity on chest pain and life interference in patients with NCCP.

## 2. Methods

### 2.1. Study Population

The investigation complies with the principles outlined in the Declaration of Helsinki. The study was approved by the local ethics committee and all participants gave written informed consent before participating.

This study was designed as a case-control study. The study consisted of 59 consecutive outpatients referred for evaluation of chest pain by the stress test at the Marmara University Hospital Department of Cardiology from May 2012 to June 2012. The fact that the referring physician had evaluated the patient's chest pain as sufficiently suspect to necessitate referral for investigation by a cardiologist was taken as adequate for study inclusion. NCCP was defined as a previous study [2, 8]. Eligibility criteria included (a) being at least 18 years of age, (b) chief complaint of chest discomfort or angina equivalent, and (c) no cardiac abnormality after a complete cardiac evaluation including general physical exam, electrocardiography, echocardiography, exercise tolerance test, myocardial perfusion scintigraphy, or coronary angiography. To augment generalizability, patients were excluded because of (a) current or lifetime cardiac diagnosis (e.g., CAD, MI), (b) current or recent diagnosed malignancies, and (c) current use of psychotropic medications or medications significantly affecting pain, a history of drug or alcohol abuse within the past six months, pregnancy, or severe psychopathology (i.e., suicidal patients, severe depression, and psychosis). Eight patients were excluded from the study because of exclusion criteria. A final sample of 51 patients participated in this study. The control group consisted of 51 healthy volunteers who were working in the Marmara University Education and Research Hospital without any cardiac disease.

### 2.2. Study Protocol

Research assistants identified eligible chest pain patients from cardiology outpatient medical records. The evaluation interview was administered to eligible patients and to control group who consented to participate. The interview included questions about sociodemographic, medical characteristics and psychiatric status. Patients and controls also completed self-reported scales which include health anxiety inventory (HAI), somatosensory amplification scale (SAS), and Toronto alexithymia scale (TAS). The research assistants were trained in the administration of the structured diagnostic interview for mental disorders and supervised by an experienced psychiatrist. The evaluation interviews were audio recorded and 25% of interviews were randomly selected for review to measure interrater agreement on psychiatric status.

### 2.3. Sociodemographic and Diagnostic Interview

This brief interview gathered sociodemographic data and general medical history (e.g., personal and family history and current medication). Hypertension was defined as systolic blood pressure ≥140 mmHg, diastolic blood pressure ≥90 mmHg, previously diagnosed hypertension, or use of any antihypertensive medications. Diabetes mellitus was defined as fasting plasma glucose levels more than 126 mg/dL in multiple measurements, previously diagnosed diabetes mellitus or use of antidiabetic medications such as oral antidiabetic agents and insulin. Hyperlipidemia was defined as serum total cholesterol ≥240 mg/dL, serum triglyceride ≥200 mg/dL, low-density lipoprotein cholesterol ≥130 mg/dL, previously diagnosed hyperlipidemia, or use of lipid-lowering medication. Smoking status was defined as the history of tobacco use at admission or in the 6 months prior to visit. Structured Clinical Interview for Diagnostic (SCID-I) and Statistical Manual of Mental Disorders, Fourth Edition (DSM-IV) [20], is a diagnostic interview recommended for the evaluation of psychiatric disorders for research purposes. It was available in Turkish.

### 2.4. Self-Report Questionnaires

The 18-item self-report HAI measures the intensity of health anxiety especially from the cognitive and emotional perspectives. This easy-to-use self-report scale can be used in patients with medical disorders as well as psychiatric patients. HAI has fourteen items to enquire psychological status of the patients (first part, HAI-1) and its last four items to carry out the data about psychology of the patients if they have a serious illness (second part, HAI-2). HAI-T score is the total score of the HAI-1 and HAI-2 subscales. All items are rated on a 0- to 3-point Likert scale and ratings are summed to produce a total score that ranges from 0 to 54. The Turkish version of HAI has adequate reliability and validity in clinical and nonclinical samples (Cronbach's alpha = 0.918).

The 10-item self-report SAS measures how the physical symptoms are experienced by patients and their susceptibility to the somatization. This scale is applicable to the patients with psychiatric or medical disorders and also to the healthy community. All items are rated on a 1- to 5-point Likert scale and ratings are summed to produce a total score that ranges from 10 to 50. The Turkish version of SAS has adequate reliability and validity in clinical and nonclinical samples (Cronbach's alpha = 0.62–0.76) [21].

Alexithymia was measured using the 20-item self-report TAS [22]. All items are rated on a five-point Likert scale, with five negatively scored items. Factor analyses have confirmed three factor subscales representing difficulty identifying feelings (DIF; and distinguishing between feelings and somatic sensations), difficulty describing feelings (DDF), and externally oriented thinking (EOT) [23]. The Turkish version of TAS-total scale and the three subscales have demonstrated adequate reliability and validity in both clinical and nonclinical populations (Cronbach's alpha = 0.85) [22, 24].

### 2.5. Statistical Analyses

Statistical analyses were performed using SPSS 16.0 statistical package for Windows. Distribution of data was assessed by using one-sample Kolmogorov-Smirnov test. Continuous data were expressed as mean ± standard deviation while categorical data were presented as a number and percentage of patients. Chi-square test was used for comparison of categorical variables. Student's *t*-test and the Mann-Whitney *U*-test were used for comparison of parametric and nonparametric variables between patient and control groups. Significance level was set at *P* < 0.05.

## 3. Results

Sociodemographic characteristics for the two groups are presented in Table 1. There were no significant differences with respect to age, sex, and education level between the patient and control groups. There were statistically significant differences in marital status, smoking, and working status. The prevalence of married people in the NCCP patient group was nearly double of controls (80.4% versus 43.1% resp.) (*P* < 0.001). The prevalence of smokers in patient and control groups was estimated to be 41.2% and 5.9%, respectively (*P* < 0.001). Control group had significantly higher rates of working status than those of patient group (*P* < 0.001). There were no significant differences in cardiac risk factors associated with cardiovascular disease like hypertension, hyperlipidemia, and diabetes mellitus. The patient and control groups were compatible with each other according to the medical history.

Table 2 presents detailed results for measures of psychological distress. There were significant differences in scores of HAI and SAS. The patient group had significantly higher scores on the SAS (*P* < 0.001), HAI-T (*P* < 0.05), and HAI-1 (*P* = 0.006) scales. As indicated in Table 3, male and female patients did differ on somatosensory sensitivity and health anxiety levels. Females reported significantly higher overall somatosensory sensitivity than males on SAS (*P* = 0.002) and higher health anxiety level on HAI-1 (*P* = 0.036) and HAI-T (*P* = 0.039) scales.  Table 4 demonstrates distributions of TAS subscale scores with respect to the patient and healthy control groups. There were significant differences in all TAS subscale scores between the two groups. Patient group reported higher on difficulty identifying feelings (DIF) (*P* = 0.016), difficulty describing feelings (DDF) (*P* = 0.005), and externally oriented thinking (EOT) (*P* = 0.002). As indicated in Table 5, patients who had total TAS score more than 50 also presented higher levels of health anxiety (*P* = 0.045). There was no relationship between alexithymia and health anxiety levels in control group.

## 4. Discussion

Being consistent with our first hypothesis, health anxiety and somatosensory sensitivity were associated with higher referral to the cardiology clinics for NCCP. Somatosensory sensitivity and health anxiety level, as anticipated, was positively associated with healthcare utilization and life interference from chest pain [8]; however, analyses separated by gender revealed that this association was more significant for women. In this study, patients with NCCP had significantly lower levels of working status. Because of bodily vigilance and cardiac anxiety on chest pain, these patients may be unable to perform correctly at work and physical sensations may have significant impact on their daily life activities [10, 15, 18, 19].

Our first findings largely congruent with theoretical models of NCCP show that personality and emotional factors are important in this medically unexplained syndrome [15, 23, 25]. As we all know nicotine addiction and difficulty of the smoking cessation therapies depend partly on genetic and environmental factors as well as psychopharmacological effects of nicotine. Also, the other probable factor mediating smoking maintenance is temperament and personality characteristics. So, nearly seven times higher rates of smoking status in our NCCP patients could be a consequence of these sensitive, less resilient, and vulnerable personalities. The prevalence of married people among NCCP patients is also nearly double of controls. Temperament and characteristic traits may have an important effect on attachment style which mediates the interpersonal relations and so marriage [25, 26]. Further research on this area needed to be conducted in order to elicit this specific issue.

Depression and anxiety are common in patients with CAD. In a recent study, we demonstrated a significant relationship between depression and anxiety scores and CAD while cardiovascular risk factors were similar between patient and control groups [27]. Psychological comorbidity has been shown to be common in NCCP as well as CAD and affects up to 75% of patients. Many studies reported a high prevalence (>50%) of anxiety disorders (including panic disorder, obsessive-compulsive disorder, and phobic disorders) and depression. Other psychological abnormalities have also been reported covering neuroticism, hypochondriac behavior, and somatization [2]. Lantinga et al. [26] found that patients with NCCP had higher levels of neuroticism and psychiatric comorbidity before and after cardiac catheterization that did patients with CAD. Anxiety sensitivity and depression influence reports of pain and thus contribute to the pathophysiology of NCCP. Extending beyond anxiety sensitivity, the present study supports that alexithymia may be a clinically relevant correlate of the NCCP syndrome. Alexithymia, originally conceptualized to explain a characteristic of patients with psychosomatic illnesses, is a multifaceted personality construct characterized by deficits in the cognitive processing and regulation of emotions [28].

Our results suggest that health anxiety sensitivity, somatosensory sensitivity, and alexithymia may be important psychological factors in the etiology and maintenance of NCCP. Within the alexithymia texture, greater difficulty identifying and describing feelings were associated with higher health anxiety. This finding is consistent with other empirical research demonstrating that NCCP individuals, who misperceive or are unable to verbally express affect-related bodily sensations and therefore report fewer or less intense emotional experiences, report more severe cardiac related physical sensations [13, 17]. Some researchers and clinicians have suggested that NCCP patients develop hypersensitivity to physical sensations (especially chest pain, shortness of breath, and palpitations) that they perceive as threatening [15]. NCCP patients who interpret chest pain as dangerous may also be likely to seek medical attention for that pain. In a recent multivariate analysis, the authors were able to develop a predictive model for distinguishing between NCCP and CAD that includes alexithymia, quality of life, and coping based on religion and seeking medical help (85.4% sensitivity and 80.0% specificity). NCCP patients with psychological disorders show diminished quality of life, more frequent chest pain, and less treatment satisfaction than NCCP patients without psychological comorbidity [22].

These results need replication in light of study limitations. In the meantime few studies have particularly examined patients with NCCP, and the present study contributes to the growing body of literature on this disorder. However, relatively small sample size and the use of a specific patient population may limit the generalizability of these findings. This study is also limited by the possibility of shared method variance. As these results relied on self-report data, this may be especially problematic for conclusions concerning alexithymia. Since alexithymia implies limited awareness of internal psychology, the appropriateness of assessing it through self-report measure may be somewhat paradoxical; that is, in the diminished ability to identify and describe affect, individuals may have difficulty completing self-report measures [23, 25, 29]. Meanwhile Toronto alexithymia scale (TAS) represents a validated and most widely used measure of alexithymia, and future research may benefit from complementing this scale with observational or behavioral measures of alexithymia. Another limitation is that the results presented here are cross-sectional, and longitudinal studies that disentangle if somatosensory sensitivity, alexithymia, and psychiatric morbidity like relate to long-term risk in NCCP patients are needed. Prospective studies with more diverse sample are needed to examine alternative models over time.

## 5. Conclusions

The NCCP syndrome has long been frustrating for the medical community. Anxiety sensitivity and alexithymia appear to represent a specific psychological vulnerability factor that would benefit from specialized psychological interventions. Research exploring the risk and resilience factors that contribute to the syndrome of NCCP is necessary in order to identify the best ways to intervene for each individual.

## Figures and Tables

**Table 1:** Sociodemographic characteristics and clinical data of study population.

	Patient group (*n* = 51)	Control group (*n* = 51)	*P*
Age (years)	33.0 ± 7.3	30.6 ± 10.6	0.195
Male *n* (%)	14 (27.5%)	20 (39.2%)	0.294
Married *n* (%)	41 (80.4%)	22 (43.1%)	**<0.001**
Literate *n* (%)	47 (92.2%)	51 (100%)	0.118
Working *n* (%)	24 (47.1%)	44 (86.3%)	**<0.001**
Smoking *n* (%)	21 (41.2%)	3 (5.9%)	**<0.001**
Hypertension *n* (%)	7 (13.7%)	1 (2%)	0.060
Diabetes Mellitus *n* (%)	3 (5.9%)	1 (2%)	0.617
Hyperlipidemia *n* (%)	6 (11.8%)	1 (2%)	0.112

Data are presented as mean ± standard deviation or number and percentage of patients.

**Table 2:** Somatosensory amplification scale and health related anxiety scores of study population.

Psychological tests	Patients group (*n* = 51)	Control group (*n* = 51)	*P*
SAS score	32.4 ± 7.5	26.8 ± 7.0	**<0.001**
HAI-1 score	14.5 ± 6.3	11.2 ± 5.4	**0.006**
HAI-2 score	3.2 ± 2.2	3.5 ± 2.2	0.442
HAI-T score	17.6 ± 7.7	14.7 ± 6.4	**0.038**

Data are presented as mean ± standard deviation.

SAS: Somatosensory Amplification Scale; HAI: Health Anxiety Inventory; T: Total.

**Table 3:** Somatosensory amplification scale and health anxiety scores between males and females.

	Patient group			Control group		
	Male	Female	*P*	Male	Female	*P*
SAS	27.0 ± 5.1	34.4 ± 7.3	**0.002**	25.1 ± 7.0	27.9 ± 6.8	0.160
HAI-1	11.5 ± 5.6	15.6 ± 6.2	**0.036**	9.4 ± 6.0	12.2 ± 4.7	0.072
HAI-2	2.7 ± 2.1	3.3 ± 2.3	0.375	3.2 ± 2.4	3.7 ± 2.0	0.386
HAI-T	14.2 ± 6.6	19.0 ± 7.8	**0.039**	12.6 ± 7.2	15.9 ± 5.6	0.070

Data are presented as mean ± standard deviation.

SAS: Somatosensory Amplification Scale; HAI: Health Anxiety Inventory; T: Total.

**Table 4:** TAS sub-scale scores distribution of the study population.

	Patient group (*n* = 51)	Control group (*n* = 51)	*P*
DIF	17.0 ± 4.2	14.8 ± 4.9	**0.016**
DDF	14.7 ± 2.9	12.9 ± 3.2	**0.005**
EOT	23.7 ± 2.9	21.7 ± 3.5	**0.002**

Data are presented as mean ± standard deviation.

TAS: Toronto alexithymia scale; DIF: Difficulty identifying feelings; DDF: Difficulty describing feelings; EOT: Externally oriented thinking.

**Table 5:** Comparison of the TAS-T and HAI-T score between patient and control group.

	TAS-T ≥ 50	TAS-T < 50	*P*
Patient group	18.7 ± 8.0	13.0 ± 3.2	**0.045**
Control group	15.7 ± 5.9	13.7 ± 6.7	0.274

Data are presented as mean ± standard deviation.

TAS: Toronto alexithymia scale; HAI: Health Anxiety Inventory; T: Total.
